# Recent Advances in Conductive Hydrogels for Electronic Skin and Healthcare Monitoring

**DOI:** 10.3390/bios15070463

**Published:** 2025-07-18

**Authors:** Yan Zhu, Baojin Chen, Yiming Liu, Tiantian Tan, Bowen Gao, Lijun Lu, Pengcheng Zhu, Yanchao Mao

**Affiliations:** Key Laboratory of Materials Physics of Ministry of Education, School of Physics, Zhengzhou University, Zhengzhou 450001, China; zy_2024@gs.zzu.edu.cn (Y.Z.); bjchen@gs.zzu.edu.cn (B.C.); lymphysic2005@stu.zzu.edu.cn (Y.L.); czzyj2000@gs.zzu.edu.cn (T.T.); wzz@gs.zzu.edu.cn (B.G.); lulijun@zzu.edu.cn (L.L.)

**Keywords:** conductive hydrogels, E-skin, electrophysiological signal, healthcare monitoring

## Abstract

In recent decades, flexible electronics have witnessed remarkable advancements in multiple fields, encompassing wearable electronics, human–machine interfaces (HMI), clinical diagnosis, and treatment, etc. Nevertheless, conventional rigid electronic devices are fundamentally constrained by their inherent non-stretchability and poor conformability, limitations that substantially impede their practical applications. In contrast, conductive hydrogels (CHs) for electronic skin (E-skin) and healthcare monitoring have attracted substantial interest owing to outstanding features, including adjustable mechanical properties, intrinsic flexibility, stretchability, transparency, and diverse functional and structural designs. Considerable efforts focus on developing CHs incorporating various conductive materials to enable multifunctional wearable sensors and flexible electrodes, such as metals, carbon, ionic liquids (ILs), MXene, etc. This review presents a comprehensive summary of the recent advancements in CHs, focusing on their classifications and practical applications. Firstly, CHs are categorized into five groups based on the nature of the conductive materials employed. These categories include polymer-based, carbon-based, metal-based, MXene-based, and ionic CHs. Secondly, the promising applications of CHs for electrophysiological signals and healthcare monitoring are discussed in detail, including electroencephalogram (EEG), electrocardiogram (ECG), electromyogram (EMG), respiratory monitoring, and motion monitoring. Finally, this review concludes with a comprehensive summary of current research progress and prospects regarding CHs in the fields of electronic skin and health monitoring applications.

## 1. Introduction

With the rapid advancement of the Internet of Things (IoT) and artificial intelligence (AI), flexible electronics have become a highly promising research domain. Their substantial potential for applications in areas such as brain–computer interfaces, neural science and technology, clinical diagnosis, and treatment has driven their growing prominence [1,2,3,4,5,6,7,8,9,10]. Notably, the scope of applications for flexible electronics has been broadened significantly, encompassing wearable sensors, soft robotics, skin patches, flexible energy harvesters, and human–machine interfaces (HMI) [11,12,13,14,15,16,17,18,19,20]. Since next-generation flexible electronics are required to withstand external forces and extreme environments, including folding, distortion, stretching, and compression, there is a growing demand for more effective material selection strategies to develop advanced flexible electronics [21,22,23,24,25,26,27,28,29]. To satisfy the features of flexible electronics, tremendous efforts have been devoted to developing a wide range of synthetic polymers, elastomers, or nanomaterials that possess both electrical conductivity and mechanical strength [30,31,32,33,34,35,36,37,38]. However, due to their intrinsic high Young’s modulus or inadequate biocompatibility, these polymer materials are difficult to match or be compatible with skin.

Owing to their simple fabrication, high tunability, biocompatibility, and soft mechanical properties, CHs have attracted extensive research interest [39,40,41,42,43,44,45,46,47]. Hydrogels represent a category of materials characterized by a three-dimensional (3D) network structure that incorporates a significant proportion of aqueous medium [48,49,50,51,52,53,54,55,56]. The high content of water endows hydrogels with biocompatibility, electrolyte conductivity, and a comfortable interface for flexible electronics [57,58,59,60,61]. Moreover, the 3D polymeric network formed through the chemical cross-linking of the polymers provides elasticity and high tunability, which are crucial for flexible electronics to adapt to various external forces and environmental conditions [62,63,64,65,66,67]. The mechanical attributes of CHs can be effectively modulated via the manipulation of their chemical structures. This approach enables the accurate tuning of stretchability, flexibility, and softness, which are essential features for developing conformal and deformable wearable devices [68,69,70,71,72,73]. Furthermore, researchers have engineered CHs with integrated functionalities, including self-adhesive, self-healing, antibacterial, freeze-resistant, and desiccation-resistant properties [74,75,76,77,78,79,80,81]. Several reviews have summarized the advancements of CHs across various fields, including materials, preparation methods, applications, and underlying mechanisms [82,83,84,85,86,87,88,89]. These comprehensive reviews offer valuable insights into understanding specific aspects of CHs, but further exploration is needed to fully address the broad applications of CHs in E-skin and healthcare monitoring.

**Figure 1:**
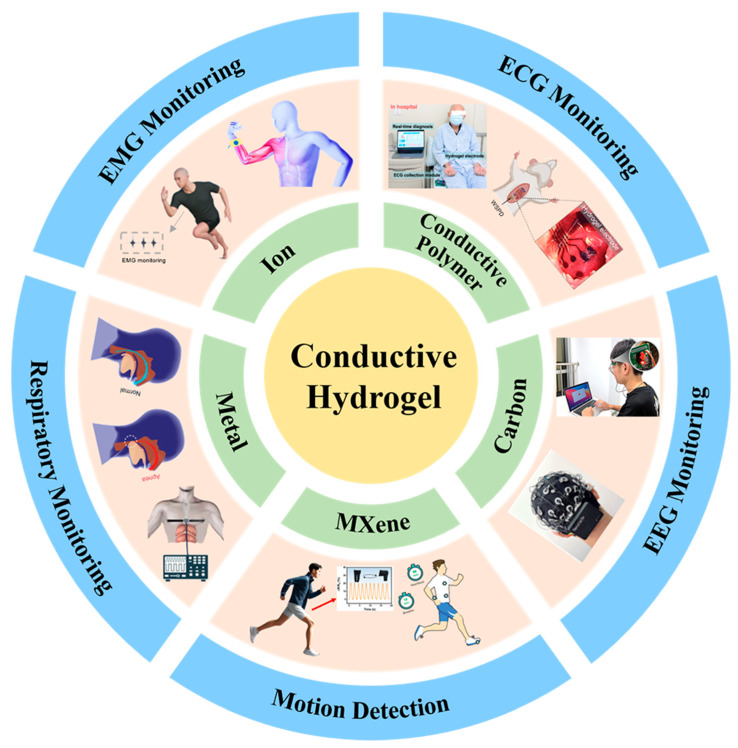
The categories and recent application advancements of CHs. The categories of CHs are mainly summarized into five parts, namely ionic CHs, polymer-based CHs, carbon-based CHs, metal-based CHs, and MXene-based CHs. Practical applications of CHs are presented, including their use in electrophysiological signal monitoring (ECG, EMG, EEG), motion detection, and respiratory monitoring [90,91,92,93,94,95,96,97,98,99]. EMG monitoring: Reproduced with permission [97]. Copyright 2025, Wiley. Reproduced with permission [95]. Copyright 2024, Wiley. ECG monitoring: Reproduced with permission [99]. Copyright 2024, Wiley. Reproduced with permission [90]. Copyright 2025, Elsevier. EEG monitoring: Reproduced with permission [92]. Copyright 2023, Wiley. Reproduced with permission [93]. Respiratory monitoring: Copyright 2024 Springer Nature. Reproduced with permission [94]. Copyright 2022, Wiley. Reproduced with permission [98]. Copyright 2025, Elsevier. Motion detection: Reproduced with permission [96]. Copyright 2025, Wiley. Reproduced with permission [91]. Copyright 2024, Wiley.

This review aims to provide a thorough summary of CHs and integrate recent progress in their applications for E-skin and healthcare monitoring. First, we summarize a systematic classification of CHs, encompassing polymer-based, carbon-based, metal-based, MXene-based, and ionic CHs, with the objective of establishing a foundational framework to help readers grasp the recent advancements in this field [90,91,92,93,94,95,96,97,98], as shown in Figure 1. Following this, we emphasize the most recent research advancements and the current status of CHs in E-skin and healthcare monitoring, including applications in electroencephalogram (EEG), electrocardiogram (ECG), electromyogram (EMG), respiratory monitoring, and motion monitoring. Finally, in light of the current development of CHs, this review summarizes the present challenges and proposes potential research trajectories for improving practical applications. We anticipate that this review will offer deeper insight into CHs and inspire further innovation in flexible electronics.

## 2. Overview of Conductive Hydrogels

Hydrogels are defined as hydrophilic 3D polymeric networks characterized by crosslinked architectures composed of hydrophilic polymer chains interpenetrated with aqueous phases. Owing to biocompatibility, tunability, and high flexibility, the integration of these hydrogels into flexible electronics has catalyzed transformative progress in the development of HMI. CHs are commonly constructed from a polymeric hydrogel matrix embedded with conductive components, in which the hydrogel network serves as a structural scaffold, affording deformability under mechanical stress and environmental stability. By incorporating conductive fillers, these CHs exhibit enhanced electrical properties, including superior charge transport capability and fast response characteristics, enabling efficient signal transduction at the bioelectronic interface. According to the charge transport mechanisms, CHs can be generally categorized into two main types, namely those relying on electron conduction and those utilizing ion conduction. The electronic conductivity of hydrogels, which typically originates from the movement of electrons via tunneling and contact mechanisms, is primarily achieved through the incorporation of highly conductive fillers such as metallic nanowires, carbon-based nanomaterials (carbon nanotubes and graphene), conductive polymers (polypyrrole (PPy), polyaniline (PANI), poly(3,4-ethylenedioxythiophene): polystyrene sulfonate (PEDOT: PSS)), and MXene. Conductive fillers play a crucial role in enhancing the performance of hydrogels by forming continuous conductive pathways within the hydrogel matrix, thereby facilitating electron transport. This synergistic interaction not only optimizes the electrical properties of the hydrogel but also improves its mechanical stability. Additionally, these fillers can promote the migration of ions, which is essential for charge transport. On the other hand, the conductive mechanism of ionic conductive hydrogel is attributed to freely mobile ions within their network, typically derived by incorporating ions or ionic groups into the polymer matrix. Based on the underlying principles of ionic conduction and recent technological progress in this rapidly developing field, ionic conductive hydrogels are principally categorized into two types, namely those incorporating conventional electrolytes and those utilizing ionic liquids (ILs). CHs integrate the unique features of conductive materials and hydrogels, thus possessing superior charge transport capability, responsive characteristics, and tunable mechanical performances. These features enable them to demonstrate enormous potential for use in health monitoring, E-skin, soft robotics, and other fields. Table 1 summarizes the electrical, mechanical, and stable properties of various material-doped CHs for electrophysiological signal and healthcare monitoring.

### 2.1. Ionic Conductive Hydrogels

Ionic conductive hydrogels, a class of polymer network materials rich in hydrated electrolytes, show great potential in applications such as flexible electronics and biomedical sensors, thanks to their unique conductivity and softness. Ionic salts in hydrogels supply many ions. When an electric field is applied, these cations and anions migrate directionally in the field to form a current. Ionic conductive hydrogels are generally fabricated through two main approaches. One is to introduce salt solutions or ILs into elastic hydrogels featuring a 3D network structure. The other is to make use of polyelectrolytes that contain repeating cationic and anionic groups [113,114,115,116]. Based on the internal mobile ions, the hydrogel becomes an excellent conductor. The 3D network structure of the hydrogel contains pores that serve as pathways for ion transport. During the last few years, the advancement of high-performance conductive materials has been driven by the increasing demand for wearable electronic devices and IoT applications [117,118,119].

**Figure 2:**
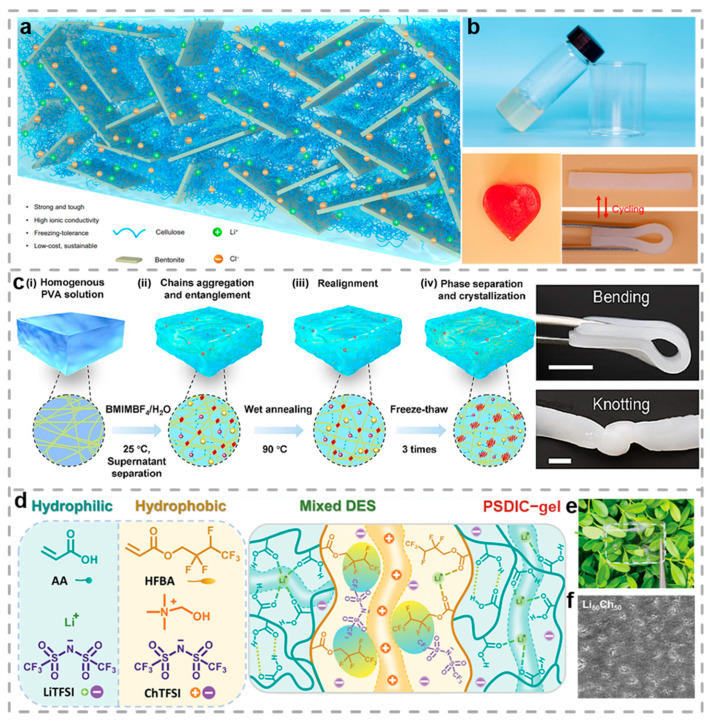
Ionic conductive hydrogels. (**a**) Illustration depicting the designed microarchitecture of cellulose/BT composite hydrogels. (**b**) Images displaying cellulose solutions and corresponding hydrogels [100]. (**c**) Diagram illustrating the fabrication methodology for ionic liquid/polyvinyl alcohol composite hydrogels [101]. (**d**) Hydrophilic deep eutectic solvent components and hydrophobic DES components. (**e**) Optical photograph of Li_50_Ch_50_ [102]. (**f**) SEM image of PSDIC-gels. All pictures have been adopted with permission.

The naturally abundant cellulose serves as an optimal candidate for the fabrication of ionic CHs. In order to enhance both mechanical strength and ionic transport in the cellulosic hydrogel, Wang et al. developed a supramolecular design approach [100]. This performance improvement stemmed from bentonite (BT) incorporation, which established strong coordination complexes with cellulose chains while creating nanoconfined interlayer spaces that effectively regulated ion transport pathways. Figure 2a shows that the cellulose–BT hydrogel contains BT nanoplatelets that form interstitial spaces acting as efficient pathways for rapid ion transport, due to their high in-plane stiffness and numerous surface bonding sites. Subsequently, the inorganic salts introduced through the immersion method notably improved the hydrogel’s freezing resistance (−30 °C to 25 °C). A functioning mechanism was proposed to explain how cellulose, BT, and LiCl work synergistically in the ionic conductive cellulose–BT hydrogel. The LiCl significantly lowers the freezing point via weakening the internal hydrogen bonding between water molecules, endowing the hydrogel with high ionic conductivity, even at subzero temperatures. As shown in Figure 2b, cellulose hydrogels with complex geometries can be readily fabricated, and those in a bent state can recover their original shape, demonstrating excellent resilience and substantial potential for adapting to diverse application scenarios.

Alternatively, owing to their negligible vapor pressure, exceptional stability under thermal and electrochemical stresses, as well as outstanding ion transport capabilities, ILs have garnered significant attention. Based on the great advantages of IL, Zhou et al. developed high-performance IL/polyvinyl alcohol (IL/PVA) hydrogels which included PVA and 1-butyl-3-methylimidazolium tetrafluoroborate (BMIMBF_4_) as a raw material [101]. As exhibited in Figure 2c, the prepared an IL/PVA hydrogel, which harnessed the synergistic properties of BMIMBF_4_ and PVA, achieved remarked tensile strength (4.99 MPa), notable toughness (2.52 MJ/m^3^), significant conductivity (31.85 mS/cm), and impressive stretchability up to 1100%, along with good fatigue resistance. The cross-linking mechanism is facilitated by the cations of the ionic liquid ([BMIM]^+^). These cations can form hydrogen bonds with the hydroxyl groups on PVA chains, thereby inducing robust interactions between the PVA chains. Furthermore, benefiting from the combined application of wet annealing and freeze–thaw cycling, the molecular chain entanglement and crystallinity of the IL/PVA hydrogels were enhanced, thereby improving their mechanical properties. The IL/PVA hydrogels exhibited promising potential for application in areas such as strain sensors and supercapacitors, offering a novel pathway for the advancement of hydrogel-based flexible electronics with multiple functionalities.

In addition, Ma et al. prepared a tough, water-resistant, and conductive eutectic gel with phase-separated dual ionic channels (PSDIC-gel) by mixing hydrophilic and hydrophobic polymerizable deep eutectic solvents and using in-suit photopolymerization-induced phase separation, as shown in Figure 2d [102]. The hydrophilic polyacrylic acid (PAA) phase is rich in Li^+^ ions, forming a conductive hard phase that provides mechanical strength and electrical conductivity. The hydrophobic poly (hexafluorobutyl acrylate) phase is rich in choline chloride cations (Ch^+^) and serves as a soft phase, which facilitates the transport of Ch^+^ ions and enhances the toughness and water resistance of the material. Moreover, the Li_50_Ch_50_ sample exhibits only a 2.5% weight loss at 200 °C by thermogravimetric analysis, demonstrating remarkable thermal stability. Among them, the Li_50_Ch_50_ samples demonstrate exceptional mechanical performance, achieving 6.03 MPa in tensile tests, 683.3% strain before fracture, and remarkable toughness (16.18 MJ/m^3^), while maintaining conductivity (1.6 × 10^−3^ S/m) through ionic transport mechanisms, with the corresponding optical photograph and scanning electron microscopy (SEM) image of Li_50_Ch_50_ shown in Figure 2d,e. This enhancement is predominantly attributed to the prevalence of hydrogen bonds and ion–dipole interactions within the polymer matrix. Additionally, Li_50_Ch_50_ demonstrates excellent water resistance and self-healing ability. After being placed at room temperature for 20 min, its mechanical property recovery rate reaches 91.9%. These properties make the PSDIC-gel well-suited for sensor applications capable of monitoring human motions, such as kneading, tapping, knocking, grasping, walking, and jumping.

### 2.2. Conductive Polymer Hydrogels

The advantageous features of both hydrogel materials and electroactive polymers are combined in conductive polymer hydrogels (CPHs). The polymer chains can provide continuous pathways for mass transfer, while the hydrogel’s 3D network structure facilitates the diffusion of both ions and electrons [120,121,122,123]. CPHs feature tunable electrochemical and mechanical properties, are light weight, and have good biocompatibility and hydrophilicity. These features position them as a promising category of polymer materials, leading to their extensive application in areas including biomedicine, energy storage, and environmental protection [124,125,126,127]. At present, many studies have shown that CPHs can be applied as flexible electrodes or electrode coatings in fields such as energy storage devices, including supercapacitors, fuel cells, HMI, and medical and healthcare. Common conductive polymers utilized in the preparation of CPHs include PANI hydrogels, PPy hydrogels, and PEDOT. This section will primarily focus on introducing the study advancements of CPHs based on these three typical conductive polymers.

**Figure 3:**
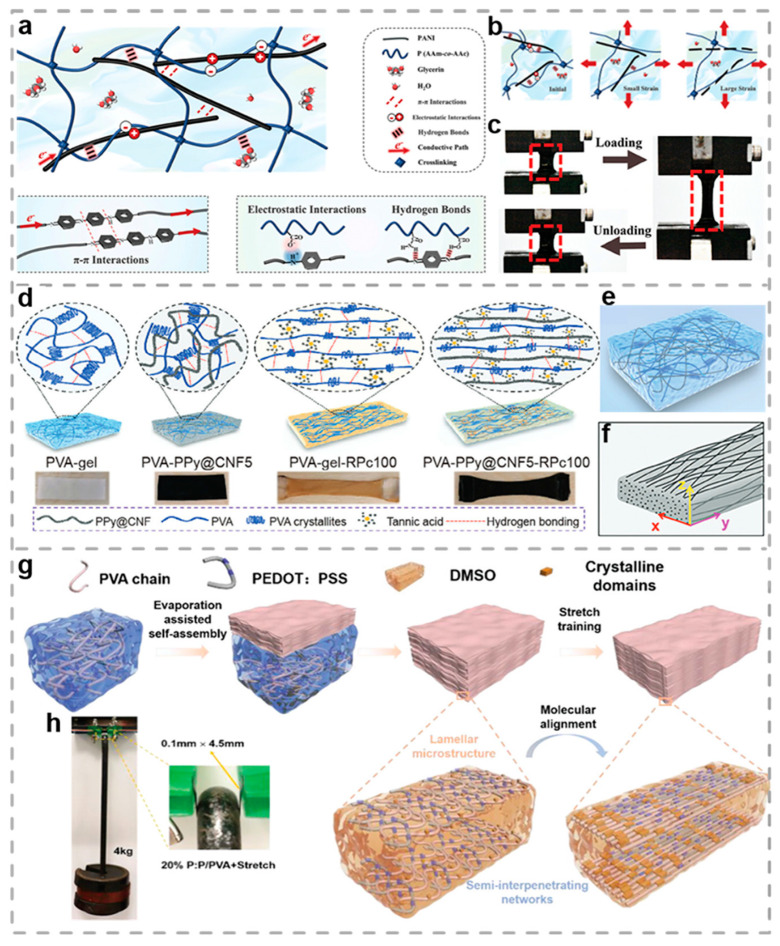
Polymer-based conductive hydrogels. (**a**) Diagrams demonstrating molecular-scale interactions within PANI/P(AAM-*co*-AAC) organo-hydrogels [103]. (**b**) Network deformation during stretching. (**c**) The corresponding snapshots of tensile loading-unloading cycles. (**d**) Schematics of structure and corresponding photographs of four types of hydrogels [104]. (**e**) The isotropic PVA-PPy@CNF hydrogel was prepared from a PVA/PPy@CNF mixture via the freezing-thawing method. (**f**) Schematic of anisotropic hydrogel morphology characterization. (**g**) Process diagram depicting the synthesis methodology for PPS-based organo-hydrogel [105]. (**h**) Optical image of PPS organo-hydrogel. All pictures have been adopted with permission.

PANI is a typical conductive polymer, whose electrical conductivity ranges from 10^−1^ to 10^3^ S/cm. It is synthesized through the chemical or electrochemical oxidative polymerization of aniline monomers. Its main chain is composed of alternating benzene rings and quinone rings, and it has reversible redox properties. Through the design of nanostructures (such as nanofibers and composites), its mechanical and electrochemical properties can be further optimized. As illustrated in Figure 3a, Chen et al. proposed an organic hydrogel with an interpenetrating network structure by threading the PANI chains through the poly(acrylamide-co-acrylic acid) network and using a glycerol/water mixed solvent [103]. Because the glycerol–water binary solvent reduces the inter-chain friction, the prepared organic hydrogel possesses high dissipated energy (1.58 MJ/m^3^) and ultra-low hysteresis (Figure 3b), demonstrating remarkable stability and durability during the 1000-cycle loading process (Figure 3c). In addition, the glycerin/water binary solvent can act as a cryoprotectant to enhance the anti-freezing performance of organohydrogels at subzero temperatures, in which the freezing temperature of organohydrogels decreases to −94.6 °C. Furthermore, the rigid PANI chains are interlaced with the flexible P(Aam-co-AAc) network via diverse noncovalent bonds, which is critical for the outstanding mechanical properties and the conductivity of organohydrogels. The organic hydrogel sensor array has been successfully applied to a robotic finger. This integration enables real-time detection of the finger’s bending angle and the pressure distribution during object grasping. In combination with a machine learning model, this system is capable of identifying the shape of the grasped object with 100% accuracy, providing strong support for the remote operation of intelligent robots and object interaction in non-visual environments.

PPy, similar to PANI, is an important conductive polymer, which is prepared by the chemical or electrochemical oxidative polymerization of pyrrole monomers. Its main chain is composed of conjugated pyrrole rings connected together, and the electrical conductivity (usually 10–10^3^ S/cm) can be significantly improved by doping with anions (such as p-toluenesulfonate, ClO_4_^−^, etc.). PPy demonstrates excellent electrochemical activity, rapid redox response, and environmental stability. Meanwhile, its morphology and properties can be optimized by adjusting the polymerization conditions (such as dopants, solvents, and temperature). In recent years, through the combination of carbon materials (graphene, carbon nanotubes) or metal oxides, the mechanical strength, cycle life, and multifunctionality of PPy have been significantly enhanced, which has promoted its development in smart wearable devices. As illustrated in Figure 3d,e, drawing inspiration from human muscle, Lin et al. designed and fabricated super strong and tough anisotropic conductive hydrogels (denoted as PVA-PPy@CNF-RPc) [104]. These hydrogels possess remarkable biocompatibility and anti-biofouling properties. The researchers selected PVA and PPy-decorated cellulose nanofibrils (PPy@CNF) as biocompatible polymers to mimic myofibers. Meanwhile, non-toxic bio-macromolecular tannic acid (TA) was employed as the mediator for the H-bonding crosslinking network, which played a crucial role in shaping the distinctive hydrogel structure. Highly entangled polymers crosslinked by crystalline domains and H-bonding endow the obtained anisotropic structured PVA-PPy@CNF-RPc hydrogel with exceptional mechanical properties. Based on these characteristics, the researchers developed a hydrogel strain sensor with multidirectional sensitivity (Figure 3f). This sensor is capable of reliably monitoring the multi-degree-of-freedom joint movements of the human body and can also control the operations of multiaxial virtual robots. Moreover, PVA-PPy@CNF-RPc hydrogel demonstrates outstanding biocompatibility and anti-biofouling properties. When triumphantly implanted into the mouse Achilles tendon, it can provide a stable electronic response signal for more than 14 days, presenting a novel solution for the application of wearable electronics.

PEDOT is a high-performance conductive polymer, which is prepared by the electrochemical or chemical oxidative polymerization of 3,4-ethylenedioxythiophene monomers. Its main chain is composed of conjugated thiophene rings and ethylenedioxy groups alternately, endowing it with high conductivity (which can reach 10^2^–10^3^ S/cm after doping), excellent environmental stability, and optical transparency. PSS is often combined with PEDOT to develop a PEDOT: PSS composite, which has the characteristics of water solubility, flexibility, and solution processability. With its low oxidation potential, high charge mobility, and biocompatibility, PEDOT finds extensive applications in diverse fields. These include organic electronics (such as OLEDs, organic solar cells, and transparent electrodes), biomedicine (neural electrodes, implantable devices), flexible electronics (strain sensors, electronic skins), and supercapacitors. Li et al. fabricated a robust and durable conductive hierarchical PVA (PEDOT: PSS/PVA) organo-hydrogel, referred to as PSS organo-hydrogel, through a straightforward combination of self-assembly and stretch training methods (Figure 3g) [105]. Benefiting from PVA/PEDOT: PSS microlayers and aligned PVA/PEDOT: PSS nanofibers, PVA and PEDOT: PSS nanocrystalline domains, and semi-interpenetrating polymer network, PSS organo-hydrogels demonstrate excellent mechanical performances (Figure 3h), such as a strength of 54.8 MPa and toughness of 153.97 MJ/m^3^. Moreover, this hydrogel also demonstrates superior mechanical and sensing characteristics. The researchers applied it to an electronic strap to track the human body’s movement signals during football training activities, including shooting, dribbling, walking, and running. The hydrogel sensor successfully demonstrated the long-term durability by subjecting it to a minimum of 200 cycles of stretching per day for a duration of 5 days. Throughout the testing period, the results consistently showed stable resistance changes, confirming the sensor’s reliability and stability. This application demonstrates the strong sensing capabilities of the PPS hydrogel under complex mechanical stress and underscores its potential for use in wearable electronic devices and HMI.

### 2.3. Carbon-Based Conductive Hydrogels

**Figure 4:**
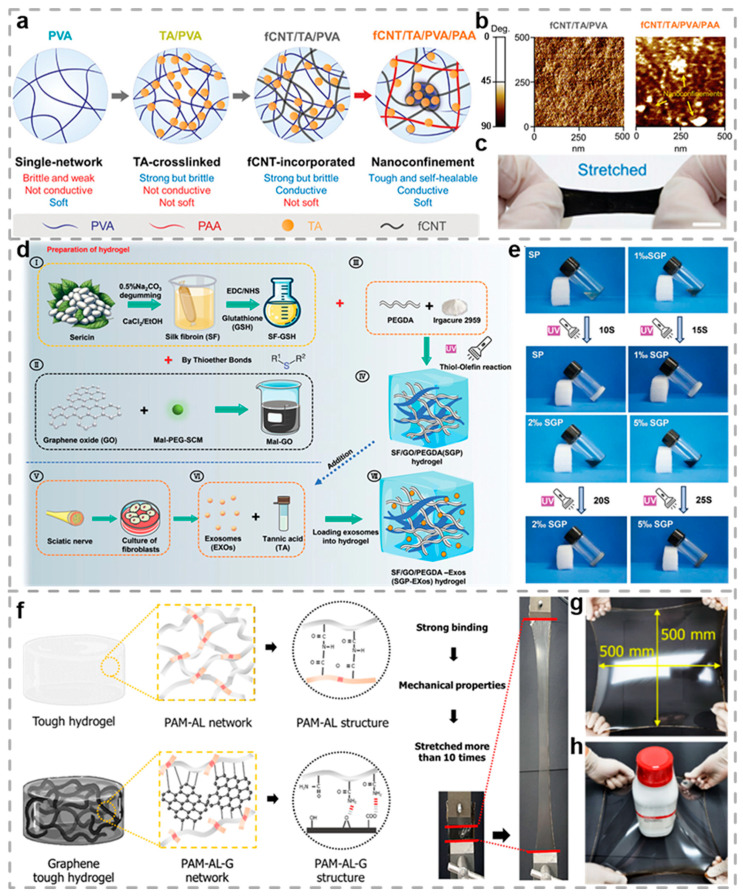
Carbon-based conductive hydrogels. (**a**) Fabrication steps of hydrogel [106]. (**b**) AFM images of fCNT/TA/PVA and fCNT/TA/PVA/PAA. (**c**) Photographs of a hydrogel under stretching. (**d**) Schematic illustrations showing the fabrication of SGP hydrogels by the three-step method [107]. (**e**) Under UV excitation, all gel groups completed the sol–gel process within 30 s. (**f**) Strategy for fabricating GTHs with highly tough, stretchable, and biocompatible properties [108]. (**g**) Photographs of GTHs under stretching. (**h**) The GTHs can be expanded up to 3000% of their original area under biaxial stretching. All pictures have been adopted with permission.

Carbon materials, including graphene, carbon nanotubes, carbon quantum dots, graphene oxide (GO), reduced graphene oxide, and others, are widely introduced into the hydrogel system to construct high-performance conductive composites [128,129,130]. Through physical blending, chemical cross-linking, or in situ polymerization strategies, carbon materials are combined with hydrogel networks (such as polyacrylamide, PVA, etc.) to create a three-dimensional interpenetrating structure, endowing materials with excellent electron/ion transport capabilities (the electrical conductivity can reach 10^−1–^10^2^ S/cm) [131,132,133,134]. Carbon-based CHs hold significant promise in various fields, including flexible electronics, energy storage (supercapacitors, battery electrodes), biomedicine (tissue engineering, electrical stimulation therapy), and environmental sensing. A non-swelling multifunctional hydrogel for bioelectronic devices was developed by Park and co-workers (Figure 4a), in which the hydrogel is composed of hydrophilic polymers PVA, carboxyl and hydroxyl-functionalized carbon nanotubes (fCNTs), TA, and PAA [106], the fCNTs and PAA through their abundant carboxyl and hydroxyl groups. This interaction forms a dynamic nanoconfinement network that endows the materials with outstanding mechanical properties, self-healing capability, and durability (Figure 4b). This hydrogel demonstrates a tissue-like modulus, high toughness, impressive stretchability, and rapid self-healing ability. The addition of fCNTs confers excellent conductivity (40 S/m) in the hydrogel network. Remarkably, this conductive performance remains stable and can fully recover following mechanical deformation or fracture, as shown in Figure 4c. Capitalizing on these superior attributes, the hydrogel has been effectively implemented in underwater EMG signal acquisition and real-time monitoring of ex vivo bladder dilation, underscoring its significant promise for advancing applications in bioelectronics.

GO has attracted substantial research attention, primarily attributed to its excellent conductivity, tunability, and high specific surface area. Benefiting from these superior characteristics, Gao et al. developed silk fibroin-based hydrogels, which were designed to promote peripheral nerve regeneration by integrating GO and fibroblast exosomes (Figure 4d) [107]. Figure 4e illustrates the sol-to-hydrogel transition. By combining silk fibroin with glutathione, then linking it with maleimide-modified GO through sulfhydryl ether bonds, and finally cross-linking it with polyethylene glycol diacrylate (PEGDA), an SF/GO/PEGDA (SGP) hydrogel is formed. This strategy not only boosts mechanical performance but also enhances electron transport ability by introducing GO, thereby providing support for the activities of nerve cells. The SGP hydrogel has excellent biocompatibility and electrical conductivity, and it can significantly facilitate the axonal extension of cells and the growth of neurons. Furthermore, by incorporating GO, Gwon and co-workers developed a robust graphene composite hydrogel (GTH) featuring nanostructures [108]. This method presents significant potential for boosting cellular affinity and mechanical robustness. Owing to the mechanical property enhancement provided by GO, the GTH demonstrates outstanding stretchability and resilience. As shown in Figure 4g, it is capable of stretching up to 20 times its original length and reverting to its initial shape once external forces are removed. The GTH, with an initial area of 81 cm^2^, can expand to over 2500 cm^2^, a deformation magnitude sufficient to cover a cylinder (Figure 4h). The GTH demonstrates excellent mechanical properties and structural integrity. On the other hand, it possesses structural and chemical characteristics conducive to cell growth and tissue regeneration. In skin defect models and tendon regeneration experiments, the TH/G 3% group significantly promoted wound healing and tendon regeneration. Moreover, the GTH demonstrates excellent electrical stability by soaking test in phosphate-buffered saline (PBS) for 7 days. After the 7-day soak, the conductivity of each hydrogel was like its initial state. The development of GTH provides new material options and research directions in the fields of tissue engineering and regenerative medicine.

### 2.4. Metal-Based Conductive Hydrogels

Over the past few years, metallic materials have been widely integrated into hydrogel systems, including metallic nanoparticles, nanowires, and metal–organic frameworks. This is primarily due to their excellent conductivity and catalytic activity, which enhance the electrical properties of the hydrogels [135,136]. Through methods such as physical doping, chemical crosslinking, or in situ synthesis, metallic components can form stable composite structures with polymer networks (e.g., polyacrylamide, sodium alginate), endowing hydrogels with high electrical conductivity (10^−1^–10^3^ S/m) [137,138,139].

**Figure 5:**
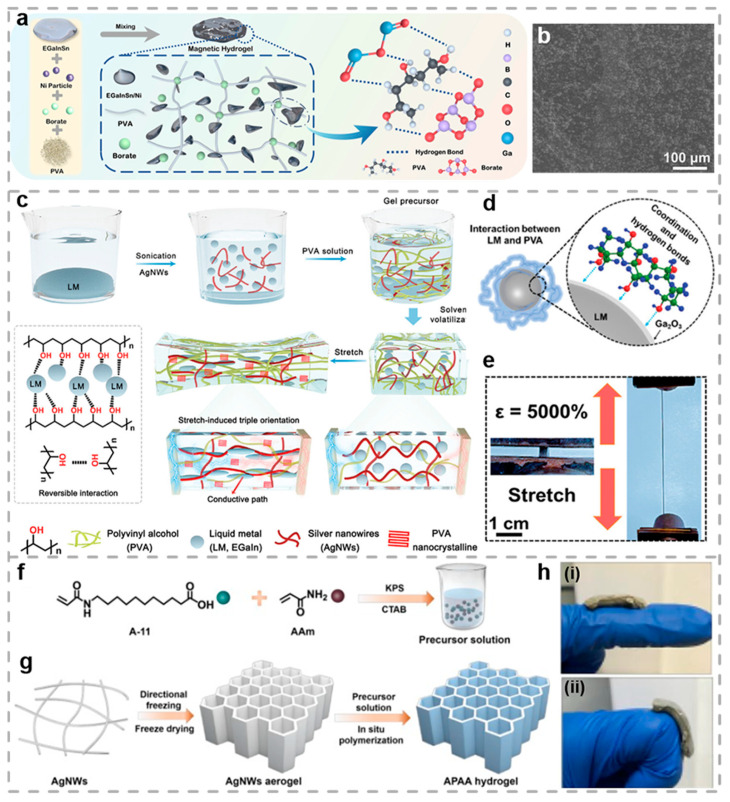
Metal-based conductive hydrogels. (**a**) Preparation of the composite hydrogel [140]. (**b**) SEM images of the composite hydrogel. (**c**) Preparation process of PAL hydrogels [109]. (**d**) Interaction between the oxide layer and PVA molecular chains. (**e**) Digital photographs of the tensile process of PAL hydrogel from 0% to 5000%. (**f**) Preparation of precursor solution and (**g**) hydrogel composite [110]. (**h**) Hydrogel as wearable sensors attached in (i) human finger and (ii) finger joint. All pictures have been adopted with permission.

The fluidity and deformability of liquid metals hold great promise for applications in a variety of fields. As demonstrated in Figure 5a, Zhao et al. proposed a composite hydrogel based on PVA/EGaInSn-Ni, with conductivity, stretchability, adaptability, biocompatibility, and self-healing capabilities [140]. Figure 5b presents the SEM characterization of the PVA/EGaInSn-Ni composite, revealing a homogeneous distribution of nickel-containing liquid metal droplets within the PVA polymer. The PVA/liquid metal hydrogel exhibits self-healing properties without external stimuli, attributed to the abundance of reversible hydrogen bonds between PVA and borate ions, along with the inherent fluidity of liquid metals, which in combination, impart this unique capability to the hydrogel. Additionally, the PVA/EGaInSn-Ni hydrogel demonstrates strain-sensitive behavior, functioning as a highly sensitive sensor capable of detecting diverse physiological movements. Its stable electrical outputs and exceptional cycling performance enable reliable monitoring of finger bending, wrist flexion, and pharyngeal contraction during swallowing. Moreover, the composite hydrogel achieves superior electromagnetic interference (EMI) shielding performance through synergistic effects between its balanced electrical conductivity and an intrinsic moisture-rich matrix. In this study, the developed hydrogel illustrates the significant potential of intelligent sensors and superior EMI. It offers a novel strategy for fabricating flexible wearable devices, which are appropriate for monitoring complex human motions.

Additionally, the inadequate compatibility between liquid metals and polymers makes it highly challenging to develop hydrogels that combine superior mechanical robustness with high electrical conductivity while maintaining stable electrical properties under tensile deformation. To address this challenge, Wang and colleagues fabricated a mechanically robust and electrically conductive hydrogel, PVA-AgNWs-LM (PAL), by incorporating AgNWs and liquid metal (Figure 5c) [109]. This strategy not only improves the hydrogels’ mechanical performance but also leads to notable enhancements in specific mechanical parameters (Figure 5d). Additionally, some studies have demonstrated that gallium can form coordination bonds with PVA, and the interactions serve to enhance the mechanical properties of the hydrogels. Specifically, within the range of 13–33 MPa, the strain can reach 3000–5300% (Figure 5e), and the toughness is improved from 390.9 to 765.1 MJ m^−3^. Impressively, when the PAL hydrogel is stretched to 4200%, the electrical conductivity increases significantly from 4.05 × 10^−3^ S m^−1^ to 24 S m^−1^, achieving a 6000-fold enhancement. This remarkable improvement is attributed to the alignment of AgNWs, compliant LM, and PVA crystalline domains, collectively establishing optimized charge transport networks throughout the hydrogel framework. In addition, the PAL hydrogel, relying on a reversible crosslinked network, enables water-induced recycling. Damaged or fatigued hydrogels can restore their initial electromechanical state through a 60 min hydrothermal treatment at 95 °C. Owing to these exceptional characteristics, the PAL hydrogel exhibits excellent performance in high-frequency signal circuits, maintaining stable signal transmission even under a large strain of 2000%, which offers new insight for next-generation reliable electronic devices.

AgNWs, a novel one-dimensional nanomaterial, exhibit superior electrical conductivity and mechanical flexibility, demonstrating substantial promise for integration into wearable electronic devices. As shown in Figure 5f,g, Huang and his team prepared a hydrogel composite with stretchability and self-healing properties by in situ polymerizing acrylamide (AAm) and N-acryloyl-11-aminoundecanoic acid (A-11) within a honeycomb-structured AgNWs aerogel [110]. Owing to reversible hydrophobic association and hydrogen-bonding interactions, the hydrogel composite exhibits remarkable EMI SE self-healing capability. The interconnected 3D silver nanowire framework enables the hydrogel to achieve high conductivity, along with a remarkably sensitive response exceeding 800%. This combination of properties enables the use of the hydrogel as a flexible motion-detection sensor for tracking human biomechanical activities (Figure 5h). The dynamic hydrophobic interactions and reversible hydrogen bonds collectively confer outstanding self-recovery properties to the hydrogel, allowing repaired material to reliably function as a motion-detection sensor. This study offers a viable approach for designing multifunctional hydrogels that simultaneously achieve superior stretchability, conductivity, and self-healing performance.

### 2.5. MXene-Based Conductive Hydrogels

As an emerging class of 2D material, MXenes have shown remarkable promise for conductive hydrogels, owing to their exceptional characteristics, including extensive specific surface area, superior charge transport capacity, hydrophilicity, and surface modifiability [141,142,143]. Due to the lamellar structure and plentiful surface functional groups, MXene can readily form multiple interactions with other materials (e.g., polymers, metal ions), thereby constructing robust conductive networks. By incorporating MXene as a nanoscale filler, it becomes feasible to simultaneously enhance the electrical and mechanical properties of the hydrogel [112,144,145,146].

**Figure 6:**
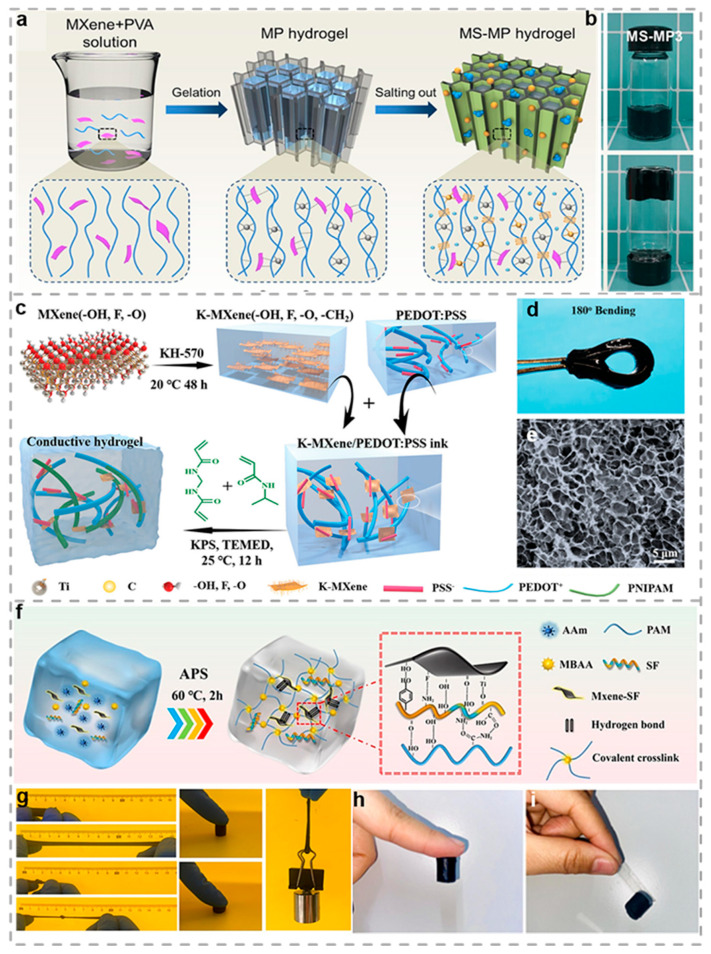
MXene-based conductive hydrogels. (**a**) Schematic of MS-MP hydrogel fabrication through the salting-out method [147]. (**b**) Digital photographs of MS-MP3 hydrogels. (**c**) Schematic diagrams depicting the synthesis of conductive hydrogels [111]. (**d**) SEM images of conductive hydrogels. (**e**) Images of the hydrogel film in bending state. (**f**) Fabrication procedure of the PAM/(MXene-SF) hydrogel [112]. (**g**) Optical images of PAM/(MXene-SF) hydrogel’s mechanical properties, including stretching, knotting, compression, and bearing a 500 g load. (**h**) Optical images of PAM/(MXene-SF) hydrogel adhesion to substrates like human finger and (**i**) PVC. All pictures have been adopted with permission.

Drawing inspiration from cellular dehydration mechanisms in electrolyte solutions, Miao et al. developed a multi-crosslinked MXene/PVA hydrogel (MS-MP) through the salting-out effect. This innovative approach enables simultaneous solid–liquid lubrication characteristics in a homogeneous system, providing a new strategy for preparing liquid-containing lubricating materials (Figure 6a) [147]. The mechanical characteristics and friction performance of the MS-MP hydrogel system can be precisely modulated through controlled variation in the salting-out intensity, enabling the synthesis of structurally robust hydrogels with optimized load support and surface lubrication properties. The MS-MP’s mechanical characteristics and friction performance can be modulated by controlling the variation in salting-out, enabling the synthesis of a structurally robust hydrogel with load-bearing capacity and lubricity. The MS-MP3 hydrogel exhibits excellent lubricating properties, with a low average coefficient of friction (0.140) and a significantly reduced wear rate, demonstrating outstanding friction reduction and wear resistance capabilities (Figure 6b). Additionally, the MS-MP hydrogel shows remarkable load-bearing capacity, withstanding compressive forces up to 300 N before failure, which is a fourfold enhancement compared to the unmodified PVA hydrogel. This work introduces a novel controllable water-release mechanism, offering a new strategy to address the challenge of hydrogel lubrication that requires external water-based lubricants.

The intricate sensing and actuation abilities displayed in biological systems have motivated researchers to develop soft robotic platforms with biomimetic tactile perception. As shown in Figure 6c, Xue et al. synthesized high-performance conductive hydrogels via in situ copolymerization of MXene/PEDOT: PSS ink with polyaniline or polypyrrole and poly(N-isopropylacrylamide) (PNIPAM) hydrogel [111]. As depicted in Figure 6d, the prepared hydrogel exhibits a uniform porous nanostructure, in which MXene nanosheets and PEDOT: PSS are homogeneously distributed. Through surface functionalization, MXene forms chemical cross-links with PANIPAM. This chemical interaction strengthens the binding between the conductive material and the polymer, enhancing the hydrogel’s stability and mechanical properties. The fabricated hydrogels display exceptional conductivity (11.76 S/m) and strain sensitivity characterized by a gauge factor of 9.93, demonstrating stable operation across 560% deformation and maintaining functionality through 300 cyclic tests at 100% strain. Moreover, the resulting hydrogel film can be repeatedly bent without sustaining any damage (as shown in Figure 6e). By further integrating these hydrogels with structural color polymers, they developed biomimetic tactile soft actuators capable of light-driven shape deformation and real-time motion monitoring. This work is anticipated to contribute novel perspectives for advanced somatosensory materials capable of simultaneous strain detection and autonomous actuation. Li and colleagues developed an innovative conductive hydrogel by incorporating silk fibroin-functionalized MXene (MXene-SF) nanosheets within a polyacrylamide (PAM) polymer matrix (Figure 6f) [112]. Experiments demonstrate that SF forms a protective layer on MXene surfaces via non-covalent interactions, which prevents MXene nanosheets from contacting water and thereby enhances their oxidative stability while inhibiting aggregation. The developed PAM/(MXene-SF) hydrogel exhibits an exceptional combination of mechanical and electrical characteristics, including excellent stretchability (1560%), skin-matched softness (E = 12 kPa), high conductivity (0.25 S m^−1^), and remarkable toughness (165 kJ m^−3^) (Figure 6g). Meanwhile, the abundant functional groups on SF macromolecules establish robust physical interactions with diverse surfaces, endowing the PAM/(MXene-SF) hydrogel with strong adhesive properties across multiple substrates (Figure 6h,i). This hydrogel electrode, with its low skin-contact impedance, enables precise detection of subtle bioelectrical signals, such as EMG and EOG, demonstrating substantial potential for detecting subtle physiological signals and implementing continuous body temperature monitoring.

## 3. Applications of Conductive Hydrogel in Healthcare Monitoring

As multifunctional materials featuring flexibility, stretchability, adhesiveness, and biocompatibility, CHs can be rapidly prepared via in situ polymerization reactions, thereby exhibiting substantial development potential in intelligent sensing, health monitoring, human motion tracking, and other emerging areas. CHs can convert external stimuli into quantifiable electrical responses through resistance or capacitance variations, permitting continuous tracking of human activities, including walking, running, respiration, and sleeping. Meanwhile, the CHs’ minimal interfacial impedance with skin enables their application as high-fidelity epidermal electrodes, capable of capturing subtle bioelectrical signals, including ECG, EMG, and EEG activity in real-time. This section centers on emerging applications of CHs for healthcare monitoring.

### 3.1. Electrophysiological Signal Monitoring

Human physiological electrical signals represent one of the most direct biophysical manifestations of life activities, with their essence originating from the spatiotemporal integration of transmembrane ion movements across cell membranes and neural electrical activities. From the ECG generated by the rhythmic beating of the heart, the EEG formed by the collective firing of brain neurons, to the EMG accompanying muscle contractions, these electrical signals carry dynamic physiological information of the human through precisely millivolt-level weak voltages. Since the emergence of electrophysiological detection techniques, the capture and analysis of physiological electrical signals have not only promoted diagnostic innovations for major medical issues such as cardiovascular and nervous system diseases but also demonstrated unique values in interdisciplinary integration within cutting-edge fields like brain–computer interfaces and intelligent prosthetic control. With breakthroughs in flexible electronics technology and AI algorithms, research on physiological electrical signals is expanding from traditional medical scenarios to emerging dimensions, including wearable health monitoring and human–machine interaction. The decoding and application of these signals have become critical technological pathways for unraveling the mysteries of life and expanding the boundaries of human capabilities. Due to their low Young’s modulus similar to biological tissues, tunable mechanical properties, and high biocompatibility, CHs have garnered extensive attention and witnessed significant advancements in their application as bioelectrical signal electrodes.

#### 3.1.1. Electroencephalogram

The EEG represents a macroscopic bioelectrical phenomenon generated by the synchronized electrical activities of hundreds of millions of neuronal populations in the cerebral cortex, which essentially reflects the dynamic integration of ionic currents induced by neuronal synaptic transmission across temporal and spatial scales [92,148]. Once recorded via scalp electrodes, these microvolt-level (μV) oscillatory signals reveal the underlying mechanisms of brain information processing, consciousness state transitions, and pathological progression, thereby providing a convenient means for diagnosing neurological disorders such as epilepsy and sleep disorders [149,150,151].

**Figure 7:**
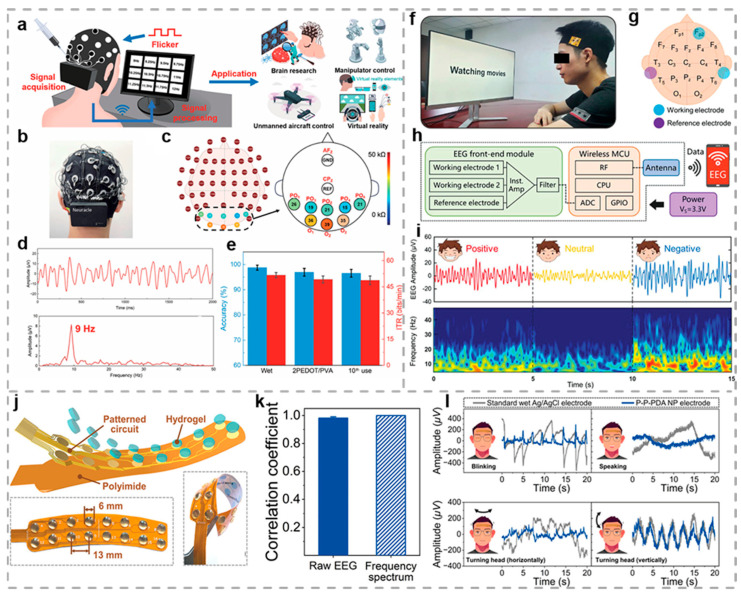
The EEG signals monitoring based on CHs electrode. (**a**) Schematic illustration of the SSVEP test [93]. (**b**) The photograph of BCI recording devices integrating PEDOT/PVA hydrogels. (**c**) Impedance after PEDOT/PVA hydrogel injection at the occipital lobe. (**d**) Power spectrum of EEG signals from Oz channel. (**e**) Comparison of accuracy and ITR between commercial electrodes and PEDOT/PVA electrodes. (**f**) Optical image of the subject viewing movie clips for EEG-based emotion induction [151]. (**g**) Schematic of EEG electrode positions on head model. (**h**) Schematic illustration of the emotion classification system. (**i**) EEG signals and corresponding energy spectra across various emotional states. (**j**) Photographs of the multichannel hydrogel electrode [92]. (**k**) Correlation of hydrogel and Ag/AgCl electrodes in EEG recording. (**l**) Motion artifact comparison of hydrogel and Ag/AgCl electrodes. All pictures have been adopted with permission.

Commercial gel-based electrodes are commonly designed for disposable application, creating a pressing requirement for reusable alternatives that can reduce long-term healthcare expenditures associated with physiological monitoring. Li and colleagues fabricated an injectable hydrogel with self-healing and reusable characteristics through the integration of PVA and borax with the concentrated PEDOT: PSS suspension (2 wt% solid content), as shown in Figure 7a [93]. Experimental measurements reveal that the PEDOT/PVA hydrogel demonstrates significantly reduced skin contact impedance (45% reduction) compared to conventional 3M Ag/AgCl electrodes in low-frequency applications, which facilitates the recording of electrophysiological signals. The PEDOT/PVA hydrogel electrodes exhibit high signal-to-noise ratios when recording ECG, EMG, and EEG signals, achieving approximately 22.1 dB, 18.9 dB, and 13.1 to 14.9 dB, respectively (Figure 7b,d). Even after multiple reuse cycles and prolonged wear (5 h), their performance remains stable, with no redness or dehydration observed on the skin. In brain–computer interface (BCI) tests, PEDOT/PVA hydrogel electrodes achieved comparable performance to standard wet electrodes, demonstrating 96.9% target identification accuracy and a 49.2 bpm information transfer rate (Figure 7e). After multiple uses and long-term storage for four months, their performance remained at a high level. The developed hydrogel electrode demonstrates significant potential for broad implementation in BCI devices and wearable bioelectronics.

EEG signals, which reflect human emotional changes and provide a viable pathway for affective recognition, suffer from low amplitude and vulnerability to interference. Current data collection devices for brain–machine interfaces typically employ rigid electrodes with multiple channels, leading to suboptimal portability and wearing comfort. Yang and colleagues engineered a dual-layer hydrogel (AHBH) interface combining adhesive and hydrophobic properties, which enables precise emotion recognition capabilities, as demonstrated in Figure 7f [151]. Drawing inspiration from the adhesion strategy of mussels and barnacles, the AHBH interface layer incorporates multiple binding mechanisms, including catechol-mediated dynamic bonds, ionic attraction forces, and hydrogen bridge formations to achieve robust interfacial adhesion. The AHBH exhibits remarkable adhesive strength (59.7 N m^−1^ on dry porcine skin and 44.1 N m^−1^ on wet porcine skin), capable of adhering firmly to various substrates, lifting a 1 kg weight, and maintaining excellent adhesiveness after 20 adhesion–peeling cycles. Researchers enhanced the AHBH through the integration of an elastic conductive composite (ECC) layer, developing an advanced biosensor capable of simultaneous perspiration monitoring, motion artifact detection, and electrophysiological signal acquisition during prolonged monitoring conditions, as evidenced in Figure 7g. Figure 7h illustrates the complete integration of AHBH-ECC biosensors with signal processing circuits and wireless Bluetooth technology into a wearable headband device designed for affective state recognition applications. Experimental outcomes reveal that the system achieves 90% mean accuracy in emotion state recognition, highlighting hydrogel interfaces’ significant applicability across multiple domains including clinical monitoring, rehabilitation training, and interactive human–machine systems.

Hydrogel-based electrodes have garnered substantial research interest due to their tissue-like mechanical performances, excellent stretchability, and biocompatibility. However, in practical applications, the electrode–tissue interface is susceptible to factors such as extreme deformation, perspiration, body movement, and vibration, resulting in reduced signal quality. Han et al. developed a flexible, self-adhesive hydrogel electrode based on PVA, polydopamine nanoparticles (PDA NPs), and polyvinylpyrrolidone (PVP) to enable prolonged wireless EEG monitoring and sustained attentiveness assessment [92]. The polymeric network comprising PVA and PVP chains confers a low elastic modulus and enhanced flexibility to the hydrogel. Simultaneously, the embedded PDA NPs improve both interfacial adhesion and electrical conductivity without compromising optical clarity. Leveraging these beneficial properties, the developed multichannel hydrogel electrodes establish uniform and reliable skin contact interfaces. These interfaces demonstrate optimal electrophysiological sensing characteristics, including frequency-stable contact impedance (3–4 kΩ across 1–100 Hz), minimal noise interference (approximately 2.85 μV), and excellent channel consistency exceeding 91% performance matching, along with perspiration resilience and motion artifact resistance, as validated in Figure 7j,k. Consequently, the electrodes are capable of recording EEG signals with exceptional quality, demonstrating minimal EEG signal fluctuations even during different movements (blinking, speaking, horizontal head turning, and vertical head turning). Then, these EEG signals are transmitted and transmitted in real time to a visualization platform via Bluetooth, as illustrated in Figure 7l. The developed hydrogel electrodes successfully classify the recorded high-fidelity frontal EEG data into seven distinct attention states, attaining a classification accuracy of 91.5%. They demonstrate excellent performance in continuous attention assessment and hold promise for advancing personal EEG monitoring technology.

#### 3.1.2. Electrocardiogram

**Figure 8:**
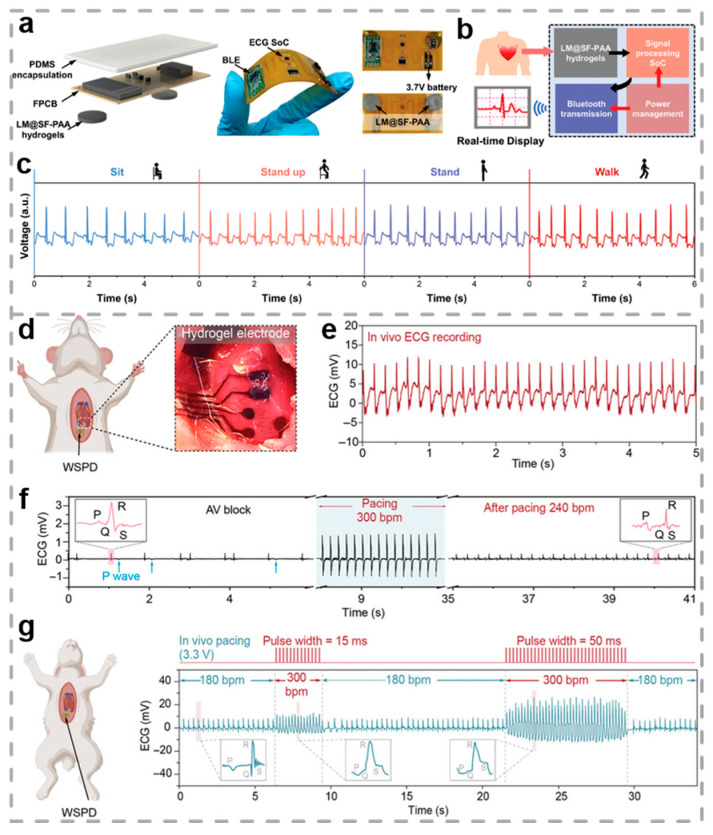
The ECG signals monitoring is based on CHs electrode. (**a**) Schematic illustration of the portable flexible ECG monitoring patch based on LM@SF-PAA hydrogel electrodes [158]. (**b**) Optical images of the ECG monitoring patch, including Bluetooth module, ECG detection, and processing system-on-chip. (**c**) ECG signals are continuously recorded by the ECG monitoring patch. (**d**) A schematic illustrates WSPD-enabled ECG recording and pacing in a rat heart [99]. (**e**) ECG signals from the rat were recorded in vivo using the WSPD system. (**f**) ECG changes in a rat model during AV block, stimulation, and post-stimulation phases. (**g**) Changes in rabbit sinus heart rate in response to stimulation with varying pulse widths. All pictures have been adopted with permission.

ECG signals arise from the electrical activity within the cardiac muscles. A typical electrocardiogram is composed of the P wave, QRS complex wave, and T wave [152,153,154]. The recording of ECG signals is crucial for the prevention, diagnosis, and treatment of cardiovascular diseases, such as myocardial infarction, valvular heart disease, and arrhythmias. [155,156,157]. Continuous ECG monitoring plays a critical role in preventing sudden cardiac emergencies, making wearable electrodes capable of efficiently acquiring ECG signals particularly important. Yan et al. fabricated a conductive hydrogel with strong adhesive properties, utilizing core–shell liquid metal@silk fibroin peptide (LM@SF) particles, with the objective of facilitating wearable and continuous ECG signal acquisition [158]. The LM@SF- PAA hydrogel exhibits mechanical properties superior to those of human skin, including high tensile strength (129 kPa), high elongation at break (1050%), low modulus (22.2 kPa), and high toughness (589 kJ·m^−3^). Based on the LM@SF-PAA hydrogel, researchers developed a portable flexible ECG monitoring patch characterized by a compact size (70 × 35 × 2 mm) and lightweight (7 g), which integrates signal acquisition, processing, and Bluetooth transmission functions to enable real-time monitoring and wireless transmission of ECG signals (Figure 8a,b). Owing to its superior adhesive and conductive properties, the LM@SF-PAA hydrogel ensures a stable physical and electrical interface with the skin during electrophysiological monitoring, thereby markedly enhancing signal acquisition quality even under conditions of motion and prolonged usage (Figure 8c). As a result, the portable ECG monitoring patch based on LM@SF-PAA hydrogel provides a new solution for health monitoring and management in telemedicine, holding significant practical value.

Traditional cardiac pacemakers rely on rigid electrode components, suffering from mechanical mismatch issues that may lead to cardiac injury and device detachment. Conductive hydrogels, characterized by their biomimetic water content, electrical conductivity, and flexibility, have become promising alternatives to traditional metallic electrodes. However, the integration of hydrogel electrodes with implantable cardiac sensing and pacing electronic systems has not yet been achieved in sensing and stimulation applications. Chang et al. developed a fully implantable wireless cardiac pacing and sensing device (WSPD) integrated with hydrogel electrodes for the treatment of arrhythmia [99]. To validate the in vivo detection and pacing functions of the WSPD system, the hydrogel was positioned on the rat’s heart surface, and ECG signals were captured using the system (Figure 8d,e). In vitro and in vivo studies confirmed that the WSPD system achieved cardiac pacing and ECG monitoring with a high SNR (≈28 dB) and stable performance (Figure 8f,g). After 31 days of implantation in rats and rabbits, the hydrogel electrodes still reliably performed cardiac pacing and ECG signal recording, showcasing excellent long-term stability. This work opens new avenues for advancing implantable flexible bioelectronic devices and shows great potential in significantly contributing to the treatment of cardiac diseases.

#### 3.1.3. Electromyogram

As functional materials integrating high biocompatibility, excellent flexibility, and tunable conductivity, CHs have demonstrated unique advantages for EMG monitoring applications over the past few years [159,160,161]. Compared with traditional rigid electrodes, CHs-based electrodes can closely adhere to dynamically deforming skin surfaces, stably capturing microvolt-level EMG signals during complex scenarios such as muscle contraction and joint movement, while mitigating mechanical irritation caused by long-term wear [95,162,163]. Furthermore, the collaborative design of functionalized hydrogels, such as self-healing, antibacterial, and environmentally responsive types, further expands their application potential in smart prosthetic control, wearable health monitoring, and human–machine interaction. This provides innovative solutions for precise, comfortable, and sustainable physiological electrical signal acquisition technologies by leveraging the multifunctional properties of CHs.

**Figure 9:**
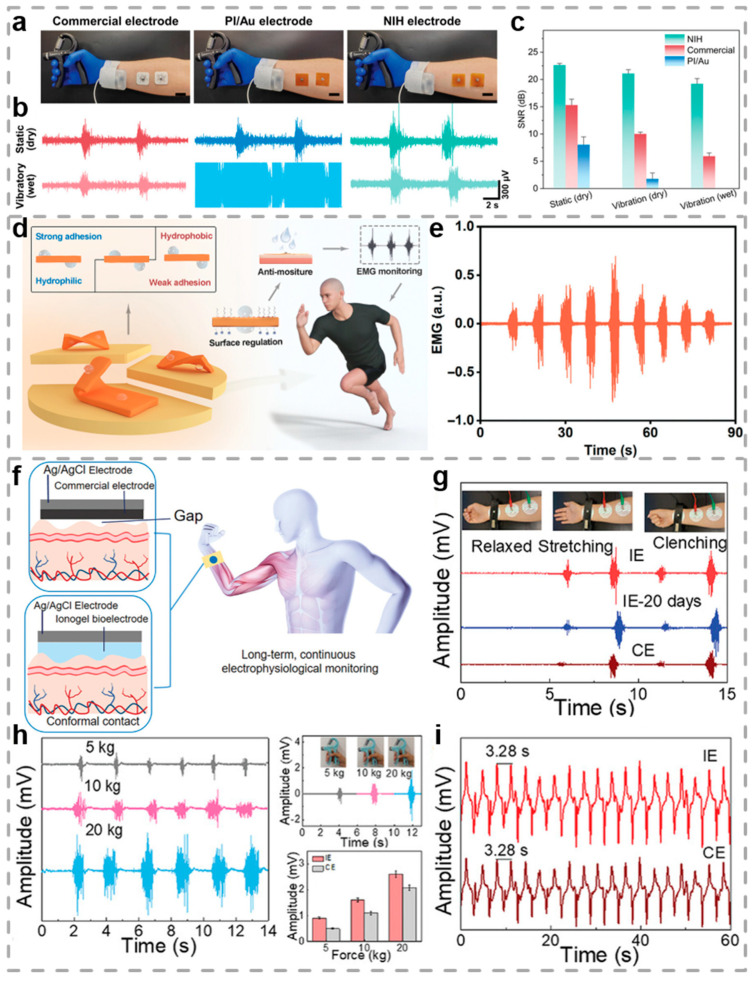
EMG signals monitoring is based on CHs electrode. (**a**) Optical photo of three different electrodes [164]. (**b**) Comparisons of sEMG signals recorded by three different electrodes. (**c**) SNR values of electrodes in static (dry skin) and vibratory (dry/wet skin) states. (**d**) Schematic of optical hydrogel surface and moisture-resistant Janus hydrogel for epidermal electronics [95]. (**e**) The raw EMG signals when the maximum load is set to 30 kg. (**f**) Schematic of P(AA-co-AU)-SC-Ga-Al^3+^ ionogel bioelectrode patch for electrophysiological monitoring [97]. (**g**) EMG signals from ionogel electrodes, commercial electrodes, and ionogel electrodes after 20 days of storage during repetitive fist clenching. (**h**) EMG signals were recorded by the ionogel electrode in different gripping forces. (**i**) Respiratory signals recorded by ionogel electrodes and commercial electrodes. All pictures have been adopted with permission.

Collecting electrophysiological signals during exercises is crucial for feedback on cardiac health and muscle injuries. Inspired by nepenthes, Yang et al. developed a wireless nepenthes-inspired hydrogel hybrid system (NIH system), which enables high-quality recording of electrophysiological signals during exercise by establishing a seamless integrated device–skin interface with fast directional sweat transport [164]. The adhesive strength of PVA/poly(acrylic acid)-based double-network hydrogels is significantly increased by more than sixfold. By further constructing nepenthes-inspired microstructures on the hydrogel surface, the directional liquid transport speed was increased 4.5-fold, thereby enhancing sweat transport efficiency. The contact impedance of commercial electrodes, PI/Au electrodes, and NIH electrodes on dry and wet skin, as well as sEMG signals and SNR recorded under different conditions, was compared. Results showed that the NIH electrode maintained high-fidelity signal recording across all conditions (Figure 9a–c). Furthermore, the NIH hybrid system is capable of real-time recording and wireless transmission of EMG waveforms and heart rate curves with a relative deviation of less than 2.6%, demonstrating its potential for precise electrophysiological signal monitoring in exercise scenarios. This achievement is expected to provide safer training support for athletes and fitness enthusiasts, while also optimizing training programs, improving performance, and reducing injury risks.

EMG, generated by muscle cell contraction and transmitted to the skin surface via ionic conductivity, holds significant application value for motion monitoring and health assessment. Wu et al. reported an interface engineering approach for synthesizing monolithic Janus hydrogels with asymmetric wettability surfaces by regulating the distribution of hydrophobic/hydrophilic monomers on the hydrogel surface, aiming at durable EMG signal monitoring (Figure 9d) [95]. The sodium dodecyl sulfate was employed as the surfactant, octadecyl acrylate as the hydrophobic monomer, and hydroxypropyl acrylate together with dimethylaminoethyl methacrylate as the hydrophilic monomers. Multiple crosslinked networks were formed via thermal radical copolymerization, denoted as SOH hydrogel. The SOH hydrogel exhibits asymmetric adhesion properties. The hydrophobic surface has a relatively weak adhesion strength, while the hydrophilic surface has a higher one. This characteristic enables the hydrogel to form a stable fit with the skin and simultaneously shows good mechanical compatibility. The Young’s modulus of this material closely matches that of the skin, enabling effective signal transmission at the skin/electrode interface. Furthermore, the SOH hydrogel exhibits excellent anti-wet adhesion capability in humid environments. In exercise-induced sweating tests, the SOH hydrogel maintains stable adhesion to the skin, whereas commercial gels swell and lose adhesiveness due to sweat absorption. With a maximum load set at 30 kg, graded loads were employed to observe changes in muscle strength, and the amplitude of EMG signals increased with increasing load (Figure 9e). This hydrogel electrode patch can durably adhere to the skin in humid environments and provide high-quality EMG signals, holding significant implications for complex application scenarios such as continuous motion monitoring, personalized digital healthcare, and multimodal human–machine interaction.

Ionogels are considered ideal candidates for constructing strain sensors to precisely detect human movements under extreme environments. Inspired by nucleobase-tackified strategies, Yan et al. prepared multifunctional adhesive ionogels via one-step radical polymerization, using acryloylated adenine/uracil and acrylic acid monomers as raw materials and sodium caseinate (SC)-stabilized liquid metal dispersions for polymerization (Figure 9f), denoted as P(AA-co-AU)-SC-Ga-Al^3+^ hydrogel. Liquid metal gallium was introduced as both a soft conductive filler and a radical polymerization initiator, while Al^3+^ served as the crosslinker [97]. The P(AA-co-AU)-SC-Ga-Al^3+^ ionogel exhibits remarkable flexibility and deformability, enduring various deformations such as stretching, twisting, and knotting. Moreover, the developed ionogel exhibits a favorable electrical conductivity (3.9 mS cm^−1^), which facilitates the efficient transformation of mechanical strain into corresponding electrical signals. By leveraging these exceptional characteristics, the P(AA-co-AU)-SC-Ga-Al^3+^ ionogel was employed in comfort-wearing epidermal sensors for real-time health monitoring of human joint motions and long-term tracking of tiny electrophysiological signals. As depicted in Figure 9g, the EMG signals generated by the brachioradialis contraction and relaxation during the repeated clenching of the fist were successfully recorded. Furthermore, the ionogel bioelectrodes can accurately capture EMG signals for grip strength and pronounced respiratory waveforms, as shown in Figure 9h,i. This work offers substantial evidence for the widespread applications of flexible electronic devices in medical and healthcare fields.

### 3.2. Respiratory Monitoring

As a core component of physiological signal monitoring, respiratory monitoring provides critical evidence for disease diagnosis, postoperative care, sleep health assessment, and exercise physiology research by real-time capturing key parameters such as respiratory frequency, amplitude, rhythm, and gas exchange [165,166]. Modern respiratory monitoring technologies are evolving toward miniaturization, intelligence, and multimodal integration. Flexible wearable sensors, including electronic skins based on hydrogels and conductive polymers, can conform to human body contours to stably acquire respiratory signals during movement or in complex environments while integrating multi-parameter monitoring functions for heart rate, body temperature, and other vital signs [167,168,169]. When combined with machine learning algorithms, these technologies further decode the correlations between abnormal respiratory patterns and pathological conditions, providing data support for early warning of respiratory diseases.

**Figure 10:**
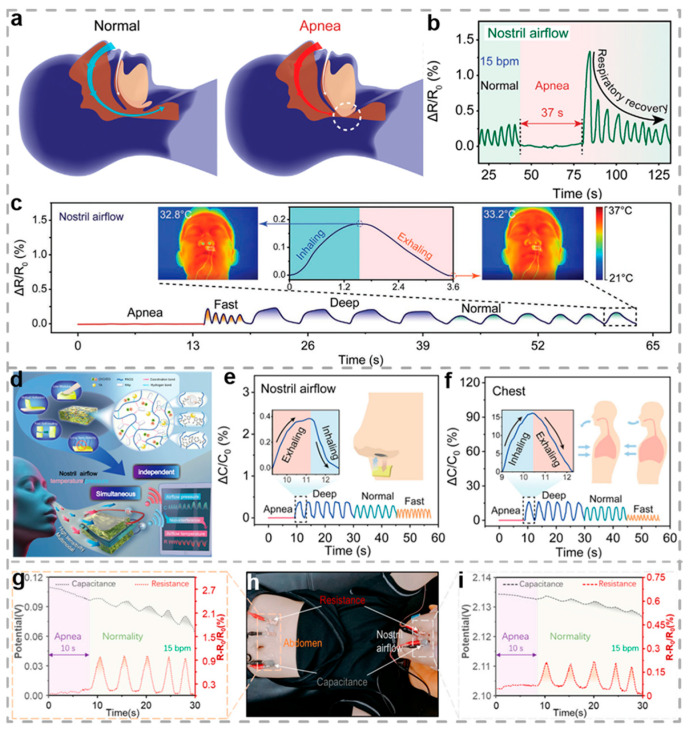
The respiratory monitoring is based on CHs electrode. (**a**) Schematic illustration of airway configurations during normal respiration and obstructive sleep apnea [94]. (**b**) Resistance fluctuations are recorded during nostril airflow monitoring; the inset presents infrared images captured throughout inhalation and exhalation. (**c**) Resistance variations from monitoring the nostril airflow, inset show the infrared images of the inhaling and exhaling process. (**d**) Schematic of ATH eutectogel structure, performance, and high-sensitivity nostril airflow sensing [170]. Capacitive variation curves representing respiratory activity monitored via nostril airflow (**e**) and chest movement (**f**). (**g**) The complete spectrum of electrical signals generated by abdominal movements [171]. (**h**) Multimodal hydrogel sensors were employed to monitor sleep apnea. (**i**) The full range of electrical signals generated by nasal airflow activity. All pictures have been adopted with permission.

Developing a respiratory monitoring system that simultaneously offers high accuracy, user comfort, ease of portability, and environmental tolerance remains a significant challenge. Liu et al. developed a multifunctional hydrogel sensor based on cellulose, intended for real-time monitoring of respiration and aiding in the diagnosis of obstructive sleep apnea syndrome (OSAS) [94]. Cellulose was selected as the structural framework of the hydrogel owing to its renewable nature, high abundance, multiple hydroxyl groups, and excellent biocompatibility. The sensor based on the cross-linked cellulose hydrogel (CH-GT) can independently monitor mechanical and thermal changes, outputting via capacitive and resistive signals, respectively. It demonstrates excellent responses to pressure changes (0–5 kPa) and temperature variations (20–100 °C). Figure 10a–c illustrate the successful implementation of the sensor for real-time respiratory monitoring and the diagnosis of OSAS. By capturing nasal airflow, thoraco-abdominal dynamics, and arterial pulse signals simultaneously, the sensor demonstrates high accuracy in identifying distinct respiratory conditions, such as normal breathing, accelerated respiration, deep inhalation, and apneic events. The hydrogel sensor significantly enhances detection accuracy and reliability through multimodal signal output and simultaneous monitoring of multiple body sites, thereby offering promising potential for wearable devices in respiratory tracking and sleep disorder prediction. Precise measurement of nasal airflow plays a vital role in real-time monitoring of respiratory activity. Nevertheless, most current approaches depend on single-mode sensing mechanisms, which are highly vulnerable to environmental disturbances and often lead to significantly reduced detection accuracy. Figure 10d shows a eutectogel-based ultrasensitive bimodal sensor developed by Liu and co-workers for real-time detection of nasal airflow pressure and temperature, applied for diagnosing OSAS [170]. Owing to the gel matrix, the optimized poly(N-acryloylglycine) composite copolymer (ATH) gel demonstrates remarkable mechanical properties and excellent self-healing capability. Moreover, the presence of multiple robust interfacial interactions between the gel and different surfaces allows ATH to form strong adhesion with various substrates, including biological tissues. A bimodal sensor was constructed by embedding a commercial elastomer (VHB 4905 3M, Saint Paul, MN, USA) as the dielectric layer between two ATH gel films. By simultaneously analyzing changes in capacitance and resistance, the device enables independent detection of pressure and temperature variations, effectively avoiding signal interference or cross-sensitivity between the two stimuli. Ultimately, the sensor was effectively utilized for real-time monitoring of respiratory behaviors and the clinical identification of OSAS. By simultaneously monitoring nasal airflow, thoraco-abdominal movements, and arterial pulse, the sensor can accurately distinguish between different respiratory states (Figure 10e,f). The bimodal sensor demonstrates significant promise for applications in personal health monitoring.

By integrating diverse sensing modalities and functions into a wearable hydrogel platform, the sensor emerges as an optimal solution for continuous, real-time health monitoring. Liu et al. proposed a breath monitoring and posture recognition system based on hydrogel for supercapacitors and multimodal wearable sensors [171]. By modifying the electrode configuration, a hydrogel sensor possessing both resistive and capacitive modes was fabricated, allowing it to sense mechanical and thermal variations through ionic conduction. In a proof-of-concept study, four hydrogel sensors were placed concurrently on selected anatomical sites to independently record nasal airflow and abdominal motion (Figure 10h), thereby assessing their diagnostic efficacy for sleep apnea. Figure 10g,i present the electrical signal profiles corresponding to abdominal motion and nasal airflow. To simulate apnea, an adult male volunteer suspended breathing for eight seconds, producing flattened signal traces that confirm episodes of airway obstruction. These results not only drive advancements in flexible electronics and sensor design but also offer novel strategies for medical health monitoring and adaptive human–machine interfaces.

### 3.3. Motion Monitoring

Over the past few years, CHs, which are a category of flexible materials characterized by bionic properties and multifunctional capabilities, have shown remarkable potential in the area of human motion monitoring [172,173,174]. Traditional rigid sensors, due to issues such as the mismatch of mechanical properties with human tissues and poor comfort during long-term wearing, struggle to fulfill the requirements for high-precision, dynamic, and continuous monitoring. CHs, by virtue of their high water content, adjustable elastic modulus, and softness similar to that of the skin, can seamlessly conform to the human body’s curved surfaces, enabling real-time monitoring of complex movements. Meanwhile, by introducing conductive fillers or ionic networks, hydrogels are capable of converting mechanical deformations into electrical signals, such as piezoresistive, capacitive, or triboelectric sensors. In addition, the incorporation of capabilities like anti-freezing, long-term stability, self-healing, and environmental responsiveness (such as dual pH/temperature responsiveness) has further expanded the applicability of CHs in extreme environments or scenarios of long-term utilization [175,176,177]. CHs are evolving from single-motion detection to multi-dimensional analysis of health status, providing a brand-new technological paradigm for personalized medicine and intelligent sports.

**Figure 11:**
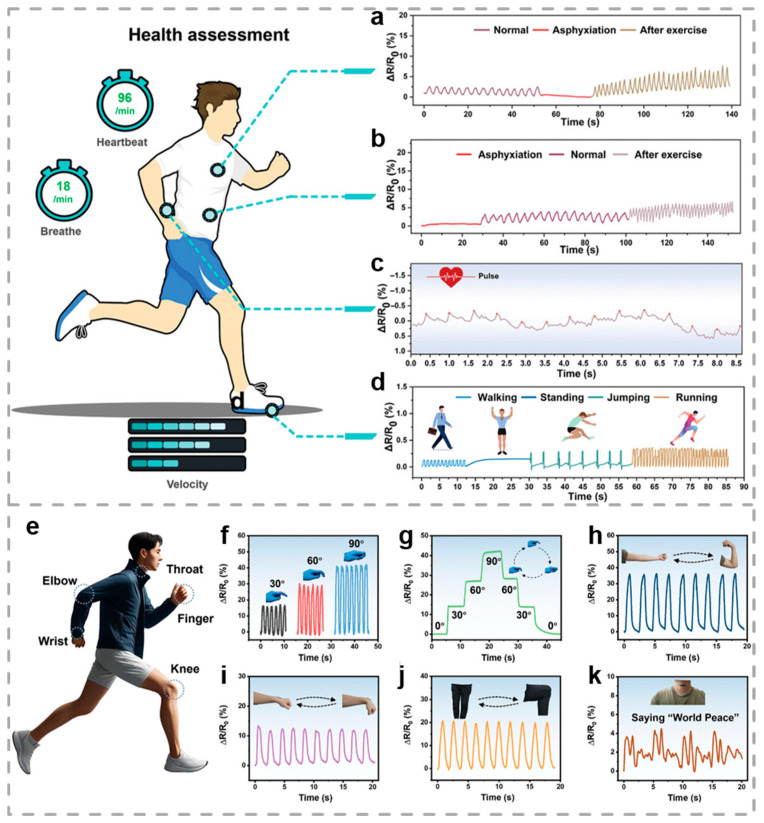
The motion monitoring is based on CHs. (**a**) Breathing monitoring is located in the chest [91]. (**b**) Breathing monitoring is in the abdomen. (**c**) The signals of pulse monitoring by strain sensor. (**d**) Recorded signals from the strain sensor during movement detection. (**e**) Human motion monitoring at five exemplified locations [96]. (**f**) Resistance variation with finger bending. (**g**) Resistance variations when holding the finger at different angles. Resistance variations with motions of the (**h**) elbow, (**i**) wrist, and (**j**) knee joints of the human body. (**k**) Resistance variation with throat motion during pronunciation. All pictures have been adopted with permission.

Conductive hydrogels derived from natural biomass, with their versatile properties, are emerging as prime contenders for applications in motion monitoring and chronic wound management. Dang and co-workers engineered a versatile hydrogel (CPPFe@TA) with multiple properties, including robust strain resistance (1560.8%), adhesiveness, self-healing capabilities, injectability, and antibacterial effects (sterilization rate of 99%) [91]. As depicted in Figure 11a,b, the CPPFe@TA hydrogel was closely attached to the chest and abdominal regions of the volunteer’s body for monitoring breathing states. As a result, the breathing states in normality, asphyxia, and post-exercise states were continuously reflected through the resistance variation in the CPPFe@TA hydrogel, demonstrating the efficiency of the hydrogel sensor in reliably identifying the magnitude and frequency of respiration. Moreover, the changes in movement or pulse can provide reference information for medical personnel to understand the patient’s disease progression and infection severity. As illustrated in Figure 11c,d, hydrogel sensors were placed on the thumb side artery of the wrist and sole of the foot, respectively, and the corresponding resistance variations in the hydrogel sensor accurately reflected pulse, walking, standing, jumping, running, and other movement states. These results demonstrate that CHs are capable of monitoring human movement and possess significant potential for future healthcare applications.

Owing to the low mechanical strength of CHs, their stability under high stress is compromised, rendering the material prone to fracture in complex or harsh environments and thereby making the simultaneous improvement of conductivity and mechanical robustness a critical challenge. Xue et al. engineered the highly robust CHs with well-aligned structures and compacted networks [96]. The hydrogels demonstrated high conductivity and sensitive sensing capability, along with outstanding mechanical performance. As illustrated in Figure 11e, a strain sensor based on hydrogel is capable of detecting multiple human body joints, such as the elbow, throat, wrist, and knee. Figure 11f,g demonstrates the strain sensor’s remarkable ability to respond to finger movement. Additionally, the strain sensor demonstrated its effectiveness in monitoring joint movement, thereby further confirming its efficacy in detecting various human motions (Figure 11h,j). The hydrogel-based strain sensor also exhibited significant promise for monitoring subtle muscle motions. As illustrated in Figure 11k, when the volunteer repeatedly spoke the words “World Peace”, similar and repetitive peaks and valleys were generated by the resistance variation in the sensor, highlighting its potential for voice recognition applications. Overall, this work offers a novel strategy for the implementation of CHs, highlighting their promising applicability in areas including flexible electronics, IoT, bioelectronics, and bionic robotics.

## 4. Conclusions and Future Perspectives

In summary, CHs represent a revolutionary variety of materials at the intersection of materials science, electronics, and biomedicine for E-skin and healthcare monitoring applications. Their outstanding properties including efficient charge transport capability, mechanical flexibility, high water content, and biological tissue compatibility have enabled significant progress in imitating the functions of human tissue and in developing non-invasive, long-term health monitoring devices. In this report, we comprehensively summarize the categories of CHs and provide a detailed introduction to the current development status and performance characteristics of various CHs. Subsequently, we comprehensively examine CHs implementation in E-skin, including electrophysiological signal monitoring (EEG, ECG, EMG) and human motion detection (monitoring of respiration and motion). In the context of E-skin, these CHs have been applied in physiological parameters monitoring (including heart rate, respiration, and muscle activity) and in facilitating regenerative processes, owing to their biocompatible nature. CHs demonstrate significant capability for precisely detecting and converting mechanical deformations, including tensile strain, applied pressure, and tactile contact, into measurable electrical responses in medical monitoring systems.

However, several challenges still impede the widespread implementation of CHs. A primary challenge lies in simultaneously optimizing three critical properties, including mechanical strength, conductivity, and multifunctionality. In many cases, enhancing one property may compromise others. Additionally, the long-term stability of CHs, especially in complex physiological or environmental conditions, needs to be further improved. For wearable and implantable hydrogel sensors, achieving excellent biocompatibility and detection sensitivity poses a critical challenge in developing safe and reliable biomedical monitoring systems. Consequently, low-cost, environmentally stable, and high-performance materials are urgently demanded to continuously improve the performance of CHs for practical applications. In the future, thanks to a unique combination of electrical conductivity, mechanical flexibility, and biocompatibility, CHs are likely to play a transformative role in fields such as flexible electronics, AI-integrated bioelectronics, and personalized medicine. The ongoing advancements in materials science and nanotechnology will continue to drive the development of CHs with enhanced performances and novel functionalities, enabling the creation of more sophisticated and efficient devices. In our opinion, while CHs have made remarkable progress in E-skin and healthcare monitoring, the careful design of molecular structures and the optimization of composition are essential to fully realize their potential and translate them into practical, reliable, and widely adopted technologies for improving human health and well-being.

## Figures and Tables

**Table 1:** Comparison of the properties of CHs based on different materials.

Materials	Conductivity (S m^−1^)	Strain (%)	Stability	Refs.
Cellulose–BT/LiCl	8.9	100	−30 °C to 25 °C	[100]
BMIMBF_4_/PVA	3.185	1000	0 °C to 317.92 °C	[101]
LiTFSI/ChTFSI/PHFBA	1.6 × 10^−3^	683	−20 °C to 200 °C	[102]
XP(Aam-*co*-AAc)	0.3	100	−18 °C to 60 °C	[103]
PVA-PPy@CNF-RPc	0.01	451.77	>14 days (in the mouse body)	[104]
PVA/PEDOT: PSS	220	400	>5 days	[105]
fCNT/TA/PVA/PAA	40	1000	>13 days (under water)	[106]
SF/GO/PEGDA	0.6	N/A	>7 days	[107]
PAM-AL-GO	N/A	2100	N/A	[108]
PVA/AgNWs/LM	24	5300	N/A	[109]
PAA/AgNWs/A-11	83	800	N/A	[110]
MXene/PEDOT: PSS/PNIPAM	11.76	560	N/A	[111]
PAM/MXene-SF	0.25	1560	22 °C to 100 °C	[112]

## Data Availability

Data sharing is not applicable.

## Abbreviations

**Full Name**	**Abbreviation**
Conductive hydrogel	CHs
Human–machine interfaces	HMI
Ionic liquids	ILs
Electroencephalogram	EEG
Electrocardiogram	ECG
Electromyogram	EMG
Polypyrrole	PPy
Polyaniline	PANI
Poly(3,4-ethylenedioxythiophene): polystyrene sulfonate	PEDOT: PSS
Bentonite	BT
Polyvinyl alcohol	PVA
1-butyl-3-methylimidazolium tetrafluoroborate	BMIMBF_4_
Polyacrylic acid	PAA
Phase-separated dual ionic channels	PSDIC
Scanning electron microscopy	SEM
Conductive polymer hydrogels	CPHs
Cellulose nanofibrils	PPy@CNF
Tannic acid	TA
Graphene oxide	GO
Functionalized carbon nanotubes	fCNTs
Polyethylene glycol diacrylate	PEGDA
SF/GO/PEGDA	SGP
Graphene composite hydrogel	GTH
Electromagnetic interference	EMI
PVA-AgNWs-LM	PAL
Acrylamide	AAm
N-acryloyl-11-aminoundecanoic acid	A-11
Poly(N-isopropylacrylamide)	PNIPAM
Silk fibroin-functionalized MXene	MXene-SF
Polyacrylamide	PAM
Brain–computer interface	BCI
Elastic conductive composite	ECC
Polyvinylpyrrolidone	PVP
Polydopamine nanoparticles	PDA NPs
Liquid metal@silk fibroin peptide	LM@SF
Sodium caseinate	SC
Obstructive sleep apnea syndrome	OSAS
